# Autophagy and Mitophagy as Essential Components of Atherosclerosis

**DOI:** 10.3390/cells10020443

**Published:** 2021-02-19

**Authors:** Anastasia V. Poznyak, Nikita G. Nikiforov, Wei-Kai Wu, Tatiana V. Kirichenko, Alexander N. Orekhov

**Affiliations:** 1 Skolkovo Innovative Center, Institute for Atherosclerosis Research, 121609 Moscow, Russia; t-gorchakova@mail.ru; 2 Centre of Collective Usage, Institute of Gene Biology, Russian Academy of Sciences, 34/5 Vavilova Street, 119334 Moscow, Russia; nikiforov.mipt@googlemail.com; 3 National Medical Research Center of Cardiology, Institute of Experimental Cardiology, 15A 3-rd Cherepkovskaya Str., 121552 Moscow, Russia; 4 Laboratory of Angiopathology, Institute of General Pathology and Pathophysiology, Russian Academy of Medical Sciences, 125315 Moscow, Russia; 5 Department of Medical Research, National Taiwan University Hospital, Taipei 10617, Taiwan; weikaiwu0115@gmail.com; 6 Institute of Human Morphology, 3 Tsyurupa Street, 117418 Moscow, Russia

**Keywords:** autophagy, mitophagy, atherosclerosis, mitochondrial dysfunction, cardiovascular disease, mitochondria

## Abstract

Cardiovascular disease (CVD) is one of the greatest health problems affecting people worldwide. Atherosclerosis, in turn, is one of the most common causes of cardiovascular disease. Due to the high mortality rate from cardiovascular diseases, prevention and treatment at the earliest stages become especially important. This requires developing a deep understanding of the mechanisms underlying the development of atherosclerosis. It is well-known that atherogenesis is a complex multi-component process that includes lipid metabolism disorders, inflammation, oxidative stress, autophagy disorders and mitochondrial dysfunction. Autophagy is a cellular control mechanism that is critical to maintaining health and survival. One of the specific forms of autophagy is mitophagy, which aims to control and remove defective mitochondria from the cell. Particularly defective mitophagy has been shown to be associated with atherogenesis. In this review, we consider the role of autophagy, focusing on a special type of it—mitophagy—in the context of its role in the development of atherosclerosis.

## 1. Autophagy

Autophagy was identified over 50 years ago as a mechanism responsible for the sequestration and degradation of cytosolic components via the lysosomal pathway. During this process, the cellular material that needs to be degraded is engulfed in a double-membrane vesicle (autophagosome) and then fuses with a lysosome to degrade it [1]. After the discovery, autophagy was believed to be a nonspecific process in which cytosolic material was randomly sequestered. However, this hypothesis was wrong. Autophagy has also been described as a selective mechanism capable of targeting viruses and bacteria (xenophagy), portions of the endoplasmic reticulum (reticulophagy), peroxisomes (pexophagy) and mitochondria (mitophagy) [2].

The mechanism of autophagy is the sequestration of substrates into two membrane vesicles for subsequent degradation. Such vesicles are called autophagosomes. The formation of a phagophore (an insulating membrane) is the initiating event of autophagy. Phagophore is formed from intracellular membrane materials. By closing, the phagophore provides complete isolation of the components inside it. The next stage is the formation of autolysosomes by fusion of autophagosomes and lysosomes, inside which the substrate is degraded due to the hydrolytic action of the lysosome [3].

Autophagy plays an essential housekeeping role in removing defective and unnecessary cell components. The role of autophagy in the health of the heart and vasculature was evaluated by regulating its activity in cardiac or vascular cell types by various triggers or inhibitors. Thus, a significant decline in cardiovascular health is linked with lowered mitophagy activity, and the activation of mitophagy was observed to be linked with refined functions of the heart and vasculature [4].

## 2. Defective Autophagy Alters the Cardiovascular Homeostasis

Numerous studies have demonstrated the importance of autophagy for the normal functioning of the cardiovascular system. The effect of altered autophagy on both cardiac and vasculature homeostasis is also well investigated [5,6,7].

For example, the deficiency of autophagy-related gene 5 (Atg5) in a mouse model leads to the sarcoplasmic accumulation of cellular waste in the form of membranous sheaths and cisternae due to the autophagy impairment. Premature cellular senescence was also observed in Atg5-deficient animals, which, in turn, is linked with mitochondrial dysfunction, myofibrillar disarray and an early deterioration in heart function. Moreover, elimination of Atg5 can cause dilated heart failure with such features as decreased ejection fraction and maladaptive remodeling (for example, hypertrophy and fibrosis), or premature death at 10 months of age [8].

Also, the essential role of lysosome-associated membrane protein-2 (LAMP-2) total elimination was observed. This protein is crucial for the lysosomal fusion with the autophagosome leading to its contents degradation, but the deficiency of the LAMP-2 can cause the premature death of mice on days 20–40 of their lives. Notably, no cardiac abnormalities are observed in Lamp2-deficient mice at a young age, but those who survive to older age exhibit severe cardiomyopathy with excessive hypertrophy and contractile dysfunction. This is probably linked with a substantial accumulation of undigested autophagosomes in the cardiomyocytes [9].

Promoting the autophagosome accumulation in young Lamp2-deficient mice by alternate-day fasting triggers the deterioration in the function of the heart [10]. That allows the suggestion that the blocked autophagic flux can be a cause of the observed cardiomyopathy when they reach old age. Taken together, these data indicate that the long-term myocardial homeostasis itself, and in response to fasting, is related to the integrity of autophagy-lysosome machinery. So, people with a mutation in Lamp2 are vulnerable to cardiac and also skeletal myopathies because of the Danon disease, which relates to glycogen storage [11].

It is also important to mention here another pathology of the cardiovascular system—chronic ischemia of the myocardium. This condition is characterized by a chronic dysfunctional but viable myocardium and results from the gradual reduction of coronary perfusion to the left ventricular myocardium. It is accompanied by an alteration in intracellular Ca2+ homeostasis and functional mitochondrial reorganization allowing mitochondria to buffer cytosolic Ca2+ and maintain the calcium-transporting capacity up to a point of irreversibility. When isolated after reperfusion, mitochondria were shown to be damaged at the structural level, and excessive production of oxygen free radicals was also observed. Damaged mitochondria undergo dramatic changes including fusion and fragmentation. AMPK (adenosine monophosphate-activated protein kinase) activation could induce autophagy, which launches cardioprotective effects against ischemia/reperfusion [12,13].

Interestingly, even limited to a single cell type, inhibition of autophagy in embryogenesis can influence the development of relevant organs [14]. Further studies are needed to describe the influence of all autophagy-related genes during mammalian cardiovascular morphogenesis. For now, we have no comprehensive data on the origin of cardiovascular abnormalities in the established constitutive knock-out models, and we cannot exclude the morphogenetic role of autophagy specific genes. Taking this into account, the best approach to investigating the involvement of autophagy in keeping cardiovascular health can be a postnatal autophagy inhibition that does not alter the development of the cardiovascular system. Another approach potentially consists of the partial diminishing of autophagy via regulating the molecular pathways that control this housekeeping process.

One of these pathways is mTOR (mammalian target of rapamycin) signaling. This signaling can be modulated in the laboratory, which allows the creation of representative animal models for investigations. For example, Gsk3a (glycogen synthase kinase-3a) knock-out in mice models diminishes autophagy via the upregulation of mTOR signaling. These mice are observed with accelerated cardiac aging even in the middle of their life. Autophagosomes accumulation in cardiomyocytes with the deficit of Gsk3a is linked to the damaged mitochondria and sarcomeres, which contributes to the development of cardiac dysfunction, myocardial hypertrophy and fibrosis. These cardiac abnormalities further aggravate with age, resulting in heart failure and a shorter lifespan [15].

Cardiac function can be restored by the use of the mTOR inhibitor everolimus, which also repairs the autophagy in Gsk3a knock-out mice. This allows avoidance of the pleiotropic side effects of Gsk3a deletion to cause the appearing phenotype [15]. Tuberous sclerosis complex 2 (TSC2) is another known modulator of autophagy. TSC2 participates in a target of rapamycin complex 1 (mTORC1) signaling pathway, which, in turn, is known to inhibit autophagy. In experiments involving cardiac-specific TSC2-deficient (TSC2-/-) mice autophagic flux was inhibited but the level of phosphorylation of S6 or eukaryotic initiation factor 4E -binding protein 1, downstream of mTORC1, was enhanced. Notably, such an effect was shown to be alleviated by the treatment with trehalose [16]. Trehalose is a natural disaccharide sugar that can be found in various organisms from bacteria to invertebrates. This compound is involved in the protection from different stress conditions such as oxidation, heat and others, due to its ability to prevent protein denaturation. Considering autophagy, trehalose was shown to induce autophagy in an mTOR- independent manner. This was established during the investigations of LC3-II, Atg5, and other relative factors, which are linked to autophagosome structure, and also phosphorylation levels of mTOR and regulatory-associated proteins of mTOR does [17].

In the same way, vascular homeostasis can be strongly damaged by the lack of non-redundant autophagy gene by any of the cell types. For example, mice who lack autophagy-related gene 7 (Atg7) in the vascular smooth muscle cells (VSMCs) demonstrate abnormal vascular contractility and calcium homeostasis [18]. The specific knock-out of Atg7 or Atg5 in endothelial cells can cause enhanced bleeding tendency due to attenuated endothelial supply of von Willebrand factor, which is a hemostatic binding protein required for platelet adhesion [19].

Significant endothelial dysfunction can be a result of the partial inhibition of autophagy within the vasculature. This can be observed, for example, by the inhibition of autophagy in response to removing transcriptional regulator Kruppel-like transcription factor-4 (KLF4). The consequence of this is impaired in vivo response to the vasodilator acetylcholine, which is a hallmark of the dysfunction of the endothelium [20]. Moreover, the vascular distensibility was shown to be attenuated by removing AMP-activated protein kinase alpha subunit (PRKAA) causing the decreased endothelial autophagic activity. The cause and effect relationship between intact autophagy and vascular functionality is further proven by the restoring of the autophagy function and, consequently, improving the functional deficit via rapamycin [21]. These data indicate the crucial role of autophagy in keeping the homeostasis of the whole circulatory system, including the heart and its vascular tree [22].

Taken together, all data mentioned in this section contribute to the importance of autophagy for cardiovascular health, for both cardiac and vascular homeostasis. Although, further investigations are needed to clarify exact mechanisms and relationships between autophagy and other cellular processes.

## 3. Autophagy in Atherosclerosis

Atherosclerosis is a chronic condition, the hallmark of which is the formation of plaques on the arterial wall. Both lipid metabolism alterations and inflammation are believed to be involved in the pathogenesis of atherosclerosis. Numerous risk factors contribute to disease development such as smoking, aging, diabetes mellitus, hypertension and high cholesterol levels [23,24].

A significant narrowing of the arteries is caused by the interlocking between increased vascular smooth muscle cells (VSMCs) proliferation, lipid accumulation, calcification, matrix turnover, and inflammation due to the plaque accumulation inside the artery lumen. Investigations in mouse models and on human specimens revealed the autophagic markers p62 and LC3-II in cells isolated from plaques and thus the dysfunctional or decreased autophagy was reported [25]. A significant lowering in LC3-II expression was revealed in patients with unstable plaques compared to patients with stable plaques [26]. This decrease would enable dead cell accumulation in the artery wall and subsequent plaque destabilization. Atheroma development was also shown to be associated with a dysfunction in autophagy. It was shown that autophagy-related gene 7 (ATG7) deletion in VSMCs [27] or a macrophage-specific ATG5-null mutant mouse model can cause mitochondrial dysfunction. In the case of ATG5-null mice, crystals of cholesterol are not deleted from plaques and stimulate enhanced production of interleukin (IL)-1β after the hyperactivation of macrophage inflammasome. Complete loss of autophagy, such as in ATG5-null cells, is lethal in both animals and humans. According to several studies, a small reduction in autophagy is not definitely linked to experimental atherosclerosis [28]. The severity of the consequences of autophagy disorders suggests that these disorders are associated with other severe dysfunctions. One of such dysfunctions can be progressive lysosomal disturbance accompanied by an accumulation of p62, which is responsible for bringing polyubiquitinated proteins to the autophagosome for lysosome-dependent degradation [29].

**Figure 1:**
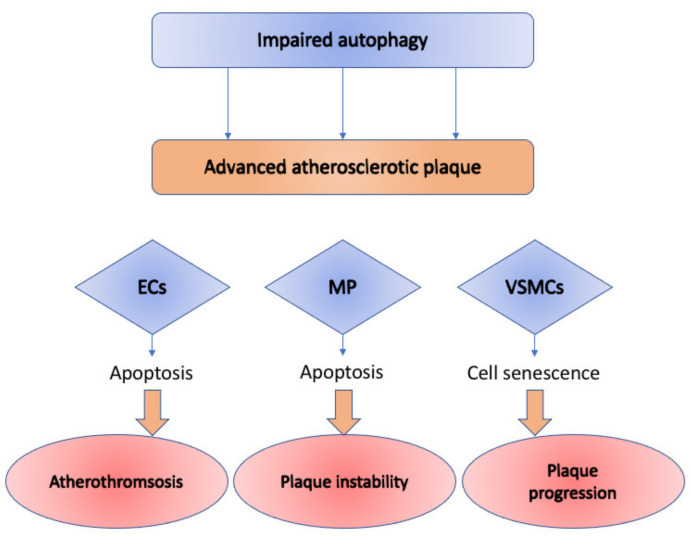
Impact of the impaired autophagy on cells of various types: ECs (endothelial cells), MP (macrophages), and VSMCs (vascular smooth muscle cells) in advanced atherosclerotic plaque. In Ecs, it causes cellular senescence and apoptosis, which leads to atherothrombosis; in MP—contributes to plaque instability through induced necrosis and apoptosis; in VSMCs—causes accelerated plaque formation through cellular senescence.

Autophagy dysfunction manifests itself in various cell types during the development of atherosclerotic plaques. Different mechanisms are involved in autophagy alterations in endothelial cells, vascular smooth cells, and macrophages [30]. The impact of the impaired autophagy on cells of various types is summarized in Figure 1.

For example, abnormal autophagy in macrophages stimulates plaque instability via induced necrosis and apoptosis [31]. Cellular senescence is stimulated by the autophagy deficiency in vascular smooth muscle cells leading to accelerated plaque formation [32]. Considering endothelial cells, decreased autophagy contributes to both senescence and apoptosis in endothelial cells [33].

It was convincingly shown that the origin of autophagy dysfunction in atherogenesis is not violated autophagosomal formation but infringed lysosomal-mediated degradation of its contents. The decline in the autophagy-lysosome system of macrophages in both human and mouse atherosclerotic plaques was identified [34].

Moreover, the damage of the lysosomal membrane can be caused by severe oxidative stress and cholesterol crystals in advanced atherosclerotic plaques, which leads to cytosolic leakage of potent acidic hydrolases [35]. The development of atherosclerotic plaques can be attenuated by trehalose or by genetically overexpressing the transcription factor EB (TFEB) through reversing the dysfunction of autophagy in plaque macrophages. Mechanistically, re-activated autophagy exerts this beneficial effect through attenuating the apoptotic and pro-inflammatory signals of plaque macrophages [34].

Taken together, these data contribute to the importance of normal autophagy for the reduction of atherosclerotic plaque burden and the protection against cardiovascular disease.

## 4. Activation of Autophagy

### 4.1. Genetic Activation of Autophagy

Several mutations were shown to improve the cardiovascular health of genetically modified mice. Data on the most well-investigated of such mutations are summarized in Table 1, as well as genetic approaches to modulation of mitophagy (see Section 8.2).

These effects can be reversed by the use of 3-methyladenine, which is an autophagy inhibitor, which once more indicated that the aforementioned cardioprotective effects can be caused by autophagy induction [36].

### 4.2. Dietary Activation of Autophagy

Another approach to autophagy activation is the reduced daily calorie intake, also called caloric restriction, which promotes autophagy efficiently in various species. Cardiovascular health was shown to be improved in various aspects by the 15%–40% reduction in caloric intake. Hypertrophy and fibrosis as well as systolic and diastolic dysfunctions in aged animals were shown to be improved by caloric restriction at the heart. The mechanism by which caloric restriction acts is the direct impact on the cardiomyocyte, including enhanced mitochondrial fitness and limited oxidative stress, apoptotic cell death, senescence, and inflammation [45].

Caloric restriction improves the function of endothelial cells and attenuates oxidative stress, collagen deposition and elastin remodeling, and thus reduces arterial stiffness [46]. It is noteworthy that these cardiovascular beneficial effects of caloric restriction manifest themselves even if applied for a short duration at a later life stage [47] and not necessarily throughout life [46]. Short-term periodic fasting was shown to stimulate autophagy and also to improve different markers of cardiovascular health, such as heart rate, systolic and diastolic blood pressure, arterial and pulse pressure, and pulse wave velocity and this supports the aforementioned concept [48]. However, caloric restriction is not always beneficial for cardiovascular health [49]. Mice that were exposed to caloric restriction showed reduced cardiac performance. Moreover, the cardiac function of such mice was even more diminished if AMP-activated kinase deletion exists, which inhibits autophagy function [50]. However, there are still many blank spots in the understanding of the relationship between diet and autophagy activation, and future investigations are still needed to clarify the exact mechanisms of this association.

### 4.3. Pharmacological Activation of Autophagy

Numerous compounds, of both natural and synthetic origin, were described to have a pro-autophagic effect and to beneficially affect cardiovascular health. For example, the natural polyamine spermidine has pleiotropic cardioprotective effects in mice and rats. Spermidine acts on cellular and molecular levels, and it can reduce or even prevent inadequate hypertrophy, myocardial stiffness diastolic abnormalities, and other signs of cardiac decline [51].

Another autophagy-stimulating compound is rapamycin, which reduces cardiac hypertrophy and loss of function in aged mice. One of the mechanisms of rapamycin action is the reduction of pro-inflammatory cytokines both on the systemic level and locally at the heart. However, it seems that only the first two weeks of rapamycin administration are effective in declining cardiac hypertrophy, as well as inducing autophagy [52].

The function of vessels in aged mice was observed to be improved by the action of the natural autophagy inducer trehalose, which acts through reducing oxidative stress and restoring nitric oxide (NO) bioavailability. These effects were also observed in humans [53].

Resveratrol, SRT1720, NAD^+^ precursors, and other sirtuins activators were shown to induce autophagy and to affect beneficially heart and vasculature health. Resveratrol decreases the signs of aging in the heart and amends vascular function in aged mice via the decrease of oxidative stress, endothelial apoptosis and inflammation [54].

These pharmaceutical compounds, also known as caloric restriction mimetics, have the potential to replace the caloric restriction itself. Preclinical testing shows that this may be a safer strategy to attenuate the decline in cardiovascular health linked to aging and potentially other risk factors [55].

## 5. Mitochondria and Mitophagy

Mitochondria are often called “energy powerhouses of cells.” The majority of cellular adenosine triphosphate (ATP) is generated from the degradation of sugars and long-chain fatty acids (FAs), and also from the metabolism of amino acids and lipids within the mitochondria. Mitochondria are also involved in Ca2+ handling and, moreover, are the key player in this process [56]. What is more, mitochondria are one of the main sources of reactive oxygen species (ROS) [57]. Due to the importance of these cellular organelles and maintaining their integrity, a special mechanism of dysfunctional mitochondria cleavage exists. Such a mechanism is called mitophagy.

If the mitochondria are damaged, it affects numerous cellular mechanisms and can violate normal cell life, even causing cell death. Various mechanisms exist that aim to keep health status and provide an efficient system of quality control. In the first mechanism, biogenesis, fission and fusion cooperate to enhance the mitochondrial population under conditions of high energetic demand or to allow a disrupted mitochondrion to fuse with a healthy organelle and substitute its violated or lost constituents [41].

Various transcription factors (TF) are involved in biogenesis, among which Transcription Factor A, Mitochondrial (TFAM), Transcription Factor B2, Mitochondrial (TFB2M), Estrogen receptor-related receptor-alpha (ERRs), Nuclear Respiratory Factor 1 (NRF1), Nuclear Factor, Erythroid 2 Like 2 (NRF2), and Peroxisome proliferator-activated receptor Gamma Coactivator 1-alpha (PGC-1α). These TFs hold the mitochondrial mass on a constant level and positively regulate other genes involved in OXPHOS, heme biosynthesis, and the import of proteins into mitochondria. Fission and fusion involves mitofusin (MFN) 1 and 2 located in the outer mitochondrial membrane (OMM) and optic atrophy 1 (OPA1) in the inner mitochondrial membrane (IMM), and other proteins mainly localized in mitochondria. These proteins are important for the mitochondrial membranes fusion, as well as exchange of mitochondrial DNA. Other proteins, fission mitochondrial 1 protein (FIS1) and dynamin-related protein 1 (DRP1), promote mitochondrial division and the passing of mitochondria to daughter cells in mitosis. The mitochondrial network is highly interconnected within healthy cells. This allows balancing of fusion and fission. Imbalanced fusion results in mitochondrial frag-mentation, and impaired fission results in mitochondrial elongation. But another mechanism is needed when mitochondrial dysfunction occurs. Such a mechanism is called mitochondrial autophagy or mitophagy [58]. For now, mitophagy is the most studied and described type of autophagy. There are various proteins and other molecules, and conditions, such as cellular differentiation, fertilization, and oxygen deprivation, are known to modulate mitophagy. Mitophagy levels were also found to vary in different human pathologies, such as cardiovascular disease (CVD), cancer, and neurodegeneration [59].

The first observation of mitophagy was made independently by two research groups. They were screening yeasts for mitophagy-deficient mutants and identified gene 32 (Atg32), which is essential for mitophagy. Aup1p and Uth1p gene products were believed to play a key role in the autophagic degradation of mitochondria, but the genomic screening has not revealed these factors [60]. Considering mammals, the maturation of reticulocytes was the first process during which mitophagy was observed. Red blood cells lose their mitochondria with the help of mitophagy during differentiation and maturation. OMM protein BCL2/Adenovirus E1B 19 KDa Protein-Interacting Protein 3-Like (BNIP3L; also known as NIP-3-Like Protein X, NIX) mediates this process with the increased expression level. BNIP3L contains a motif necessary for binding to microtubule-associated protein 1 light chain 3 (MAP1 LC3) [61], which is allocated on the autophagosomal membrane surface and intermediates the sequestration of mitochondria into autophagic vesicles.

## 6. Mechanism of Mitophagy Dysfunction

If the mitophagy mechanism is altered, a prolonged accumulation of damaged mitochondria occurs along with excessive inflammation, which is stimulated by pro-inflammatory cytokines. The importance of mitochondria, as well as mitophagy, is well-established for the transmission of innate immunity signals, and mitophagy was also demonstrated to be an essential mechanism of regulation, that limits excessive inflammation and retains tissue homeostasis.

It was further revealed that homology between Nix/BNIP3L and BCL2 Interacting Protein 3 (BNIP3) reaches 50%. Notably, it is required for autophagy, as well as for mitophagy in both cancer and during myocardial ischemia/reperfusion (I/R) injury (IRI) under hypoxic conditions [62,63]. Thus, mammalian mitophagy was proposed to be a mechanism conserved across various cell types and not only in reticulocytes. Then, elements of the mitophagy process were detected within various tissues of humans, and also a specific mechanism that manages mitophagy was revealed. This pathway was identified during Parkinson’s disease investigations [64]. A specific form of recessive Parkinsonism is marked with mutations in the Parkin RBR E3 Ubiquitin Protein Ligase (PARK2) gene, whose product is a cytosolic E3 ubiquitin ligase named Parkin, and PTEN-induced kinase 1 (PINK1), which encodes a kinase allocated on the surface of mitochondria [65].

**Figure 2:**
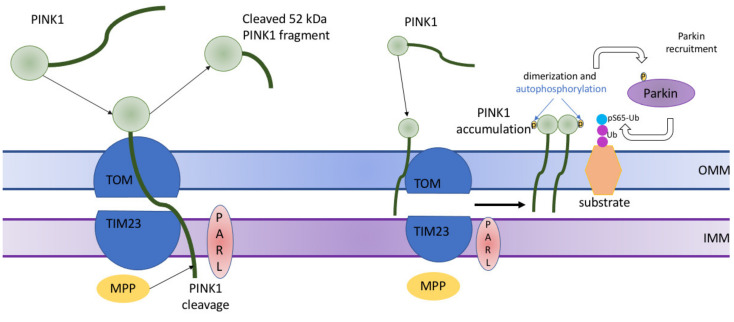
Brief description of PTEN-induced kinase 1 (PINK1)-mediated stimulation of mitophagy in normal and damaged mitochondria. In normal mitochondria (on the left), PINK1 is imported into mitochondria with the help of translocase of the outer membrane (TOM) complex and translocase of the inner membrane (TIM). At the IMM, PINK1 is initially processed by matrix processing peptidase (MPP), which removes PINK1’s N-terminal mitochondrial targeting signal, and then PINK1 is being proteolytically cleaved by the intermembrane serine protease presenilin-associated rhomboid-like protein (PARL), which cleaves the full-sized PINK1 form of 64 kDa into 60 kDa and 52 kDa fragments. In damaged mitochondria (on the right), PINK1 stores on the outer mitochondrial membrane of only injured mitochondria and is stabilized in a TOM complex (TOM7, TOM40, TOM70, TOM20, and TOM22). PINK1 mediates two various phosphorylations serving for the transformation of the autoinhibited E3-ubiquitin (Ub) ligase Parkin into an active phospho-Ub-dependent enzyme. Then, the direct phosphorylation of Parkin at S65 in the N-terminal Ubl domain occurs, increasing the activity of its E3 ligase. After that, PINK1 triggers the addition of phosphate onto S65 of Ub. This complex regulatory mechanism results in activation of the E3 ligase activity of Parkin, which allows it to ubiquitinate mitochondrial proteins through direct interaction with phospho-Ub conjugates on the mitochondria. OMM—outer mitochondrial membrane; IMM—inner mitochondrial membrane.

PINK1 is a mitochondrial serine/threonine kinase, which contributes to cell survival, especially under oxidative stress, and to normal mitochondrial functioning. Under normal conditions, PINK1 moves into the mitochondria via the activity of TOM (translocase of the outer membrane) and enters the mitochondrial inner membrane through the activity of TIM (translocase of the inner membrane). This recognizes an amino-terminal mitochondrial targeting sequence. During these events, PINK1 is being proteolytically cleaved by the intermembrane serine protease presenilin-associated rhomboid-like protein (PARL), which cleaves the full-sized PINK1 form of 64 kDa into 60 kDa and 52 kDa fragments [66]. This process is briefly shown on the left of Figure 2. After the release into the cytosol, the fragment of 52-kDa length is subsequently degraded by the proteasome [67]. In general, such mechanisms allow maintaining very low levels of PINK1 under unstressed conditions.

Damage to the population of mitochondria is associated with the full-length PINK1 mitochondrial import disruption. Then, PINK1 stores on the outer mitochondrial membrane of damaged mitochondria and the complex of TOM7, TOM20, TOM22, TOM40, and TOM70 provides its stability [68]. PINK1 acts as an intermediary in two various phosphorylation events serving for the transformation of the autoinhibited E3-ubiquitin (Ub) ligase Parkin into an active phospho-Ub-dependent enzyme. First, autophosphorylation at S402, S228, and T257 occurs. Notably, mutations in mentioned residues inhibit at the same time activity of PINK1 and Parkin accumulation at the surface of mitochondria [69]. Then, the direct phosphorylation of Parkin at S65 in the N-terminal Ubl domain occurs, increasing the activity of its E3 ligase [70]. Then, PINK1 triggers the addition of phosphate onto S65 of Ub. This complex regulatory mechanism results in activation of the E3 ligase activity of Parkin, which allows it to ubiquitinate mitochondrial proteins through direct interaction with phospho-Ub conjugates on the mitochondria. The recruitment of specific Ub-binding autophagy receptors is triggered by the presence of these poly-Ub chains to link disrupted mitochondria to LC3-positive phagosomes for clearance in lysosomes. P62/sequestosome 1 (p62/SQSTM1) was found to be the main receptor involved in mitophagy in initial investigations. Then, not less than four additional receptors, including OPTIN (optineurin), NDP52 (nuclear dot protein 52), NBR1 (neighbor of Brca1), and TBK1 (TAX1BP1) were shown to take part in the selective disposal of dysfunctional mitochondria in addition to p62. However, it is still unclear, which adaptor is effectively essential for mitophagy [71]. Studies performed with the use of cell lines with all five receptors knockout have shown that OPTIN and NDP52 are potentially crucial for mitophagy but these investigations have several limitations, including the use of the single-cell line, while not all tissues and even cell types can be compared on levels of these proteins [72].

## 7. Mitophagy in Atherosclerosis

Numerous investigations were conducted to establish the link between atherosclerosis and mitochondrial dysfunction [73]. However, the majority of such studies revealed associations, mostly with oxidative stress, clinical manifestations of atherosclerosis, and risk factors [74]. Mitochondrial dysfunction is potentially linked to arterial aging and medial degeneration [75]. This is associated with changes in the expression of genes that regulate the number of mitochondria. What is more, eliminating dysfunctional mitochondria can delay arterial aging.

**Figure 3:**
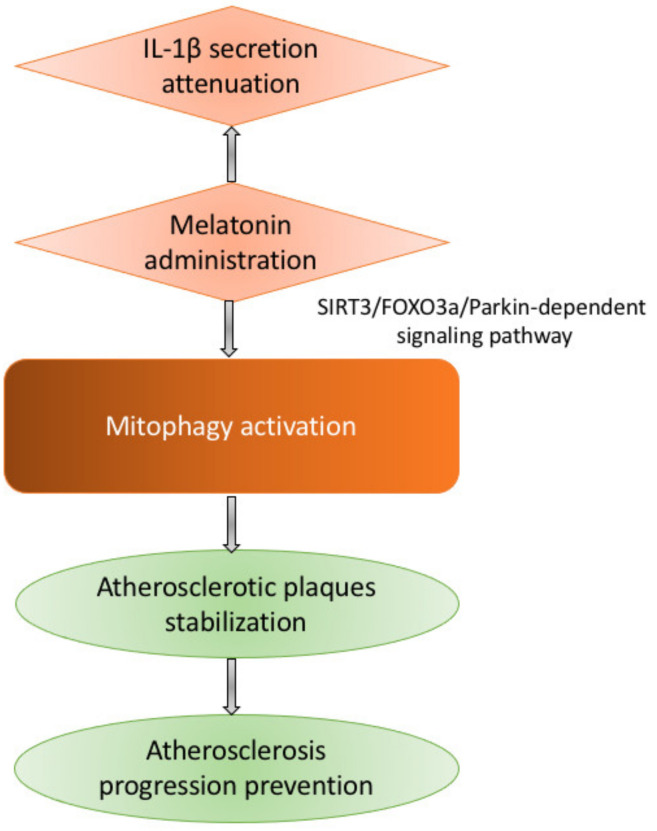
Melatonin administration affects atherosclerosis progression through an SIRT3/FOXO3a/Parkin-dependent signaling pathway. This leads to the stabilization of atherosclerotic plaques and, thus, prevents the further development of atherosclerosis. Also, melatonin reduces the IL-1β secretion.

Unfortunately, our understanding of mitophagy in the scope of atherosclerosis is far from comprehensive. It is known that inflammation, lipid metabolism alterations, and oxidative stress are the cornerstones of atherosclerosis development, and oxLDL-induced or stimulated by exogenous melatonin administration activation of mitophagy prevents the progression of the disease through stabilizing atherosclerotic plaques [76,77]. Melatonin was shown to regulate mitophagy through an SIRT3/FOXO3a/Parkin-dependent signaling pathway and to attenuate IL-1β secretion (see Figure 3).

Mitochondrial dysfunction is crucial for oxidative stress, which is an extremely important pro-atherogenic molecular mechanism. Nevertheless, it seems that oxidative metabolism is not essential for vascular ECs in which the glycolytic process is a crucial resource of energy. Mitochondria are essential for EC functioning stimulating the production of nitric acid, intracellular signaling, apoptosis, and other processes. [78]. Senescence of EC was shown to be triggered by ROS overproduction, which can stimulate apoptosis and promote atherosclerosis development [47]. In VSMCs, the function of mitochondria is inhibited by oxLDL via down-regulation of respiratory activity and production of ATP. OxLDL also triggers VSMC hyperplasia, proliferation and migration, that means the promotion of the pro-atherogenic neointima formation [79]. Notably, HSG (hyperplasia suppressor gene), which is a rodent ortholog of human fusion protein Mfn2, stops the VSMCs proliferation. Expression of HSG was observed to be strongly inhibited in affected arteries of apolipoprotein E (apoE)-deficient hypercholesterolemic mice. Reduced plaque progression and decreased VSMC proliferation were observed in a rabbit model of atherosclerosis in response to the overproduction of Mfn2, which represent the anti-atherogenic properties of human Mfn2 [80]. So, mitochondrial dysfunction affects vascular cells in opposite ways—it induces senescence and death of ECs and stimulates phenotypic switch in VSMCs from the quiescent contractile to proliferative “synthetic” phenotype.

An overexpression of PINK1 or Parkin was shown to activate mitophagy and thus to protect vascular smooth muscle cells exposed to atherogenic lipids, at least in vitro [81].

## 8. Activation of Mitophagy

Due to the great significance of mitophagy for normal cellular functioning and especially for cardiovascular health, the ways of its regulation attracted the attention of numerous researchers all around the globe. These investigations helped to identify various approaches to activate mitophagy [82,83].

### 8.1. MicroRNAs

MicroRNAs play an important role in mitophagy regulation. These are small, single-stranded non-coding RNA molecules that can prevent the target mRNAs translation and also trigger their degradation [84]. Decreased mitophagy and mitochondrial dysfunction were observed in the injury/reperfusion model of human adult cardiomyocytes, in which MiR-410 is markedly upregulated. MiR-410 was also reported to decrease the generation of ATP, mitophagy, mitochondrial membrane potential, and even cell viability if overexpressed. Downregulation of this microRNA showed an opposite effect. HSPB1 is the direct target of MiR-410, which modulates its activity and regulates mitophagy [85]. Upregulation of MiR-137 was shown under hypoxic conditions. MiR-137 downregulates Nix and FUNDC1 and impairs mitophagy [86]. In the mouse models of lipid toxicity and diabetes, MiR-133a was shown to be downregulated, and Nix was upregulated. Mitochondrial function was regulated, the potential of the mitochondrial membrane was stabilized and Nix translation was suppressed in response to upregulation of miR-133a [87].

### 8.2. Pharmacological Agents

One of the most popular drugs reported to be able to regulate mitophagy is melatonin. Melatonin prevents the opening of the mitochondrial permeability transition pore (mPTP) and inhibits the PINK1/Parkin activation within the microcirculating endothelial cells of an ischemia/reperfusion mouse model. Mitophagy-mediated cell death can also be prevented by melatonin due to the mitochondrial fission inhibition following ischemia/reperfusion injury, which restores bound VDAC1-HK2, limiting cell death in the cardiac microvasculature. The underlying mechanism is potentially related to the inhibitory effects of melatonin on mitochondrial fission VDAC1 HK2 mPTP mitophagy axis via activation of AMPKα [88]. Parkin translocation and Mst1 repression are probably involved in restoring mitophagy in diabetic cardiomyopathy by melatonin [89]. It was then reported that melatonin induced mitophagy through the Sirt3/FOXO3a/Parkin signaling pathway attenuates NLRP3 inflammasome activation, and, moreover, inhibit the atherosclerosis development [76].

Data on other pharmacological agents are summarized in Table 2.

### 8.3. Signaling Pathways

Various signaling pathways are implicated in mitophagy regulation. Thus, NR4A1 was shown to be strongly upregulated in a mouse atherosclerosis model established with ox-LDL, and also to control Parkin activation through posttranscriptional CaMKII modification. This resulted in the activation of Parkin-mediated mitophagy. The exuberant mitophagy leads to mitochondrial quality aggravation, which consequently results in mitochondrial dysfunction and energy shortage [96]. It was also reported that the blockage of the MAPK-ERK-CREB signaling pathway upregulates NR4A1 and represses Mfn2-mediated mitophagy. Damaged mitochondria are sequestered in rab5-positive early endosomes via the ESCRT mechanism in cardiomyocytes. This mechanism relies on Parkin, and the vulnerability of cardiomyocytes and embryonic fibroblasts to cell death is increased in response to the rab5 function loss [97]. P53-TIGAR axis activation was shown to downregulate BNIP3, inhibit mitophagy and also resulted in the altered mitochondria recruitment, and alleviated the cardioprotective effect in a mouse model of ischemia. The JNK signaling pathway plays an essential role in the regulation of the FOXO3a transcription factor. This stimulates the expression of BNIP3 in the models of heart failure. The mitophagy level is influenced by JNK through regulating the BNIP3 level [98].

### 8.4. Activators/Inhibitors and Gene Knock in/out

Several receptor antagonists, inhibitors of upstream molecules, and gene knockouts regulate mitophagy, directly or indirectly. Genetic approaches to modulating mitophagy are summarized in Table 1, and brief data on known modulators of mitophagy are provided below.

STAT1 is one such modulator. This protein is typically localized in the mitochondria and serves as an LC3b binding partner. LC3b, in turn, contributes to mitophagy suppression and cell death triggering in the myocardial ischemia/reperfusion response [44]. G protein-coupled estrogen receptor 1 (GPER) agonist enhances the activity of GPER and downregulates the expression of PINK1. Moreover, GPER decreases the translocation of Parkin from the cytosol to the mitochondria via inhibiting the PINK1/Parkin pathway and mitophagy. This contributes to the protection of mitochondrial structural integrity and function, and protection of the heart after ischemia/reperfusion injury [99]. Activation of ALDH2 inhibits phosphatase and PINK1/Parkin expression both in ischemia/reperfusion rats and hypoxia/reoxygenation H9C2 cells, protecting the heart against ischemia/reperfusion injury via regulating mitophagy, preventing 4-hydroxynonenal, ROS, and mitochondrial superoxide accumulation [100]. Overexpression of Sirt3 activated Fjxo3A deacetylation and Parkin expression, stimulated Parkin-dependent mitophagy, suppressed apoptosis and mitochondrial damage in cardiomyocytes, and also crucially influenced the diabetic cardiomyopathy initiation and development in a mouse model of diabetic cardiomyopathy [39]. Apelin-13 enhances mitophagy via stimulation of the PINK1/Parkin pathway in atherogenesis. F13A acts as an antagonist of apelin-13 in PINK1 and Parkin-dependent mitophagy, which suppresses the apelin-13 influence in the atherosclerosis development [101]. Mitophagy can be alleviated by the inhibition of BNIP3 activation by DUSP1 overexpression, which also inactivates the JNK pathway. This contributes to the better survival of myocardial tissue after ischemia/reperfusion [43].

### 8.5. Environmental Stimuli

The environment can also affect mitophagy in various ways and change the content of the proteins related to this phenomenon. Parkin in hippocampal neurons in a cardiac arrest model is downregulated in response to mild hypothermia, which contributes to protecting mitochondria in the neurons, improving neurological function after cardiac arrest, and diminishing mitophagy [102]. FUNDC1-dependent mitophagy activation is stimulated, and ischemia/reperfusion cardiac injury is lowered by hypoxic preconditioning [44]. The mitochondrial function of the myocardium adjusts to the stress that occurs during acute exercise. This is manifested in the mitophagy-related protein BNIP3 substantial upregulation, which, in turn, induces mitophagy and reduce myocardial injury [102]. But exercise pre-conditioning was shown to markedly upregulate parkin-dependent mitophagy and thus to inhibit exhaustive exercise-induced hypoxia-ischemia injuries.

## 9. Conclusions

Autophagy was shown to be extremely important for cardiovascular health, and the dysfunction of autophagy contributes to cardiovascular disorders, especially atherosclerosis. Autophagy dysfunction manifests itself in various cell types during the development of atherosclerotic plaques. Different mechanisms are involved in autophagy alterations in endothelial cells, vascular smooth cells and macrophages. It was convincingly shown that the origin of autophagy dysfunction in atherogenesis is not disrupted autophagosomal formation but interrupted lysosomal-mediated degradation of its cargo.

Numerous approaches to the activation of autophagy have been established. Among them, there are genetic, dietary and pharmacological ways. However, there is currently no established efficacy in atherosclerosis treatment but such approaches can be the basis for future investigations.

Mitophagy seems to be the most important type of autophagy in the scope of atherosclerosis. Mitochondrial dysfunction and defective mitophagy contribute crucially to the disease pathogenesis. There are also several different approaches to controlling mitophagy such as through miRNA, various signaling pathways and others.

It is now clear that autophagy, especially mitophagy, is essential for cardiovascular health. Mitophagy regulators also seem to be promising targets for atherosclerosis treatment.

## Figures and Tables

**Table 1:** Genetic approaches for modulation of autophagy and mitophagy along with their impact on the cardiovascular system.

Genetic Approach	Affected Process	Effects on Cardiovascular System	Reference
Mutation: dnPI3K	Autophagy stimulation	Cardiac aging attenuation; Longer lifespan; Improved cardiac functional reserve; Attenuated fibrotic and hypertrophic remodeling; Rejuvenated phenotype of cardiomyocytes; Lower lipofuscin accumulation.	[37]
Mutation: Akt2 deletion	Autophagy stimulation	Improved contractile function and cardiomyocyte calcium homeostasis in aged mice.	[36]
Parkin and PINK1 silencing	Mitophagy inhibition	Increased apoptosis in VSMCs through oxLDL	[38]
Sirt3 overexpression	Mitophagy stimulation	Activation of deacetylation of Foxo3A and expression of Parkin; Inhibition of mitochondrial damage and apoptosis in cardiomyocytes; Diabetic cardiomyopathy development in a mouse model of diabetic cardiomyopathy	[39]
Mst1 knockout	Mitophagy stimulation	Parkin upregulation; Increased mitochondrial translocation; Protection of diabetic mice myocardium	[40]
BAG3 knockdown	Mitophagy inhibition	Alteration in the clearance of defective mitochondria; Increase in levels of toxicity within the cells and subsequent cell death; Heart failure.	[41]
CsA and PINK1 knockout	Mitophagy inhibition	Downregulation of PINK1 and Parkin in senescent cardiomyocytes; Prevention of cardiomyocyte senescence	[42]
Akt2 Knockout	Mitophagy stimulation	Upregulation of Foxo1-related BNIP3, PINK1, and Parkin; Maintaining of mitochondrial integrity; Cardiac aging prevention	[36]
DUSP overexpression	Mitophagy inhibition	Inactivation of JNK pathway; better survival of myocardial tissue after ischemia/reperfusion	[43]
SWI/SNF deletion	Mitophagy stimulation	Formation of small and fragmented mitochondria	[44]
FUNDC1 knockout	Mitophagy inhibition	Aggravation of cardiac injury in the I/R model	[44]

**Table 2:** Pharmacological agents affecting mitophagy.

Agent	Effect on Mitophagy	Effects on Cardiovascular System	Reference
Melatonin	Activation	Opening of the mPTP prevention; inhibition of the PINK1/Parkin activation within the microcirculating endothelial cells of an i/r mouse model; prevention of mitophagy-mediated cell death; attenuation of NLRP3 inflammasome activation; inhibition of atherosclerosis development;	[76,88]
Simvastatin	Activation	Reduction of infarct area in mouse model of myocardial infarction; mTOR signaling inhibition in mice model and HL-1 cells; Stimulation of mitochondrial translocation of Parkin and p62/SQSTM1	[90]
Liraglutide	Activation	Oxidative stress reduction; redox reaction balance; SIRT1 and Parkin upregulation; mitochondrial homeostasis maintaining	[91]
Zinc (Zn)	Activation	PINK1 and Beclin1 upregulation; Prevention of superoxide generation; Prevention of mitochondrial membrane potential loss during reperfusion; mitochondrial oxidative stress inhibition; Cardioprotection	[92]
TEMPOL	Activation	Upregulation of PINK1 and Parkin; Promotion of cardiac recovery in aging animals.	[93]
Curcumin	Inhibition	Suppression of BNIP3 effects.	[93]
Erythorbic acid	Inhibition	Lowering the mitochondrial injury and necrotic cell death of cardiac myocytes; Suppression of BNIP3 effects; Improvement of oxidative damage; Improvement of cardiac dysfunction.	[94]
Naringin	Inhibition	Inhibition of Parkin translocation to the mitochondria.	[95]

## Data Availability

Not applicable.
